# Overdiagnosis and Overtreatment in Prostate Cancer

**DOI:** 10.3390/diseases13060167

**Published:** 2025-05-24

**Authors:** Zaure Dushimova, Yerbolat Iztleuov, Gulnar Chingayeva, Abay Shepetov, Nagima Mustapayeva, Oxana Shatkovskaya, Marat Pashimov, Timur Saliev

**Affiliations:** 1 Department of Fundamental Medicine, Al-Farabi Kazakh National University, Almaty 050040, Kazakhstan; zaure.dushimova@kaznu.edu.kz; 2 NJSC “Marat Ospanov West Kazakhstan Medical University”, Aktobe 030019, Kazakhstan; 3 S.D. Asfendiyarov Kazakh National Medical University, Tole Bi Street 94, Almaty 050000, Kazakhstan; 4 National Research Oncology Center, Astana 020000, Kazakhstan; 5 JSC “Research Institute of Cardiology and Internal Diseases”, Almaty 050000, Kazakhstan

**Keywords:** prostate cancer, PSA screening, overdiagnosis, overtreatment, cancer treatment, healthcare resources

## Abstract

Prostate cancer (PCa) is one of the most common malignancies among men worldwide. While prostate-specific antigen (PSA) screening has improved early detection, it has also led to significant challenges regarding overdiagnosis and overtreatment. Overdiagnosis involves identifying indolent tumors unlikely to affect a patient’s lifespan, while overtreatment refers to unnecessary interventions that can cause adverse effects such as urinary incontinence, erectile dysfunction, and a reduced quality of life. This review highlights contributing factors, including the limitations of PSA testing, advanced imaging techniques like multi-parametric MRI (mpMRI), medical culture, and patient expectations. The analysis emphasizes the need for refining screening protocols, integrating novel biomarkers (e.g., PCA3, TMPRSS2-ERG), and adopting conservative management strategies such as active surveillance to minimize harm. Risk-based screening and shared decision-making are critical to balancing the benefits of early detection with the risks of unnecessary treatment. Additionally, systemic healthcare factors like financial incentives and malpractice concerns exacerbate overuse. This review advocates for updated clinical guidelines and personalized approaches to optimizing patient outcomes while reducing the strain on healthcare resources. Addressing overdiagnosis and overtreatment through targeted interventions will improve the quality of life for PCa patients and enhance the efficiency of healthcare systems.

## 1. Introduction

Prostate cancer (PCa) is one of the most prevalent cancers among men worldwide. It develops in the prostate gland, a vital component of the male reproductive system [1]. Screening for PCa typically involves the prostate-specific antigen (PSA) test, digital rectal examination (DRE), and imaging studies [2]. While these screening methods have undoubtedly contributed to the early detection of PCa, they have also led to the identification of numerous indolent tumors that may never progress to clinically significant disease [3].

PCa often develops slowly, and many men may have no symptoms in the early stages of the disease. However, as the cancer progresses, some common symptoms may include difficulty urinating, frequent urination, blood in urine or semen, erectile dysfunction, pelvic pain or discomfort, and bone pain. The diagnosis of PCa usually involves a combination of medical history analysis, physical examination, blood tests, and imaging studies [4]. It should be taken into account that the modern diagnosis of PCa is associated with a number of problems: from the limitations of available screening methods to the difficulties of interpreting test results [3,5]. Despite advances in medical technology, the accurate detection of PCa remains a major challenge due to various factors. While early detection and treatment are crucial for improving survival rates, the contemporary medical landscape is increasingly grappling with the issues of overdiagnosis and overtreatment [6].

Overdiagnosis occurs when individuals are diagnosed with a condition that would not have caused symptoms or harm during their lifetime [7]. In the context of PCa, the widespread use of PSA testing has led to the detection of small, slow-growing tumors that may never progress to a stage where treatment is necessary. As a result, many men undergo unnecessary interventions, exposing them to the risks of treatment-related complications such as urinary incontinence, erectile dysfunction, and bowel dysfunction.

Several factors contribute to the overdiagnosis and overtreatment of PCa [6,8]. Advances in screening techniques, particularly PSA testing, detect many slow-growing, clinically insignificant cancers. The lack of specificity in diagnostic tools results in false positives, leading to unnecessary interventions. Medical culture and patient expectations for early detection and treatment contribute to the phenomenon, as do guidelines that historically advocated for routine screening. Moreover, economic incentives and the availability of advanced diagnostic technologies further drive overuse. Psychological factors, including fear and anxiety, also play a role in opting for aggressive treatments. Table 1 provides a comprehensive analysis by incorporating potential risks, patient outcomes, ethical considerations, and global best practices in PCa diagnosis and treatment.

## 2. Risk of Overdiagnosis and Overtreatment of Prostate Cancer

The PCa diagnosis carries the risk of overdiagnosis and overtreatment, particularly for slow-growing tumors that pose little threat, especially in older men with comorbidities. A fear of missing aggressive disease often leads to unnecessary radical treatments, causing side effects like incontinence, erectile dysfunction, and bowel issues. This harms patients and strains healthcare resources.

Overdiagnosis means identifying cancers that would not cause harm or clinical symptoms during a person’s lifetime. In the context of PCa, the widespread use of prostate-specific antigen (PSA) screening has led to the identification of many low-risk tumors that may never progress to clinically significant disease [20,21]. This phenomenon has contributed to the increased incidence of PCa and unnecessary interventions.

Overtreatment occurs when patients receive aggressive therapy for low-risk PCa that may not require immediate intervention [6]. Treatments such as radical prostatectomy, radiation therapy, and androgen deprivation can lead to serious side effects, including urinary incontinence, erectile dysfunction, and bowel dysfunction. Overtreatment can reduce patients’ quality of life and impose a significant economic burden on health care systems [22,23].

Several factors lead to the overdiagnosis and overtreatment of PCa [24]. One of the major factors contributing to overdiagnosis is the widespread use of prostate-specific antigen (PSA) testing for screening purposes. PSA testing can detect elevated levels of prostate-specific antigen in the blood, which may indicate the presence of PCa [25]. However, PSA testing is not specific for PCa and may produce false-positive results due to benign conditions such as prostate enlargement or inflammation [26,27]. As a result, many men undergo unnecessary biopsies and treatment for indolent or non-life-threatening PCa detected by PSA screening, leading to overdiagnosis. It must be understood that PSA screening, although effective in detecting diseases in the early stages, is not specific and can lead to false-positive results.

Another factor contributing to overdiagnosis is the lack of consensus on the clinical significance of screening-detected PCa. PCa covers a spectrum, from slow-growing tumors that may never cause symptoms or harm to aggressive cancers that require immediate treatment. Distinguishing between clinically significant and indolent PCa is challenging, and overdiagnosis occurs when men are diagnosed and treated for tumors that would never have progressed to a symptomatic or life-threatening stage [28].

In addition, the fear of missing a cancer diagnosis and the need for the early detection of cancer contribute to overdiagnosis. Doctors may be inclined to recommend screening and diagnostic tests, such as biopsies and imaging studies, even in cases where the likelihood of detecting clinically significant cancer is low. Patient anxiety and a desire to reassure them also play a role, leading to an increased demand for screening tests and diagnostic procedures that may not be medically necessary.

The introduction of advanced imaging techniques such as multi-parametric magnetic resonance imaging (mpMRI) has further complicated the problem of overdiagnosis [29,30]. Unlike random biopsies, mpMRI allows for the visualization and localization of suspicious lesions within the prostate, thereby enabling a more targeted approach to biopsy. Importantly, mpMRI can identify men who are unlikely to have clinically significant prostate cancer (csPCa), allowing for the safe deferral of biopsy in many cases. This selective strategy contributes to reducing unnecessary invasive procedures and the associated risks of detecting and treating indolent disease.

Robust evidence from clinical trials supports the effectiveness of mpMRI in minimizing overtreatment (Table 2). The landmark PRECISION trial [31], a multicenter randomized study involving 500 men with elevated PSA levels, demonstrated that using mpMRI as a triage test before biopsy significantly improved the detection of clinically significant cancer while reducing the diagnosis of insignificant tumors [32]. In this study, 28% of men in the MRI arm avoided biopsy altogether because their scans showed no suspicious lesions. Moreover, the detection rate of csPCa was notably higher in the MRI-targeted biopsy group (38%) compared to the standard biopsy group (26%). Importantly, the detection of clinically insignificant cancers was reduced by more than half, from 22% in the standard group to just 9% in the MRI group.

Similar findings were reported in the PROMIS trial [33], a prospective multicenter study that assessed the diagnostic performance of mpMRI compared to TRUS biopsy in 576 men [34] (Table 2). MpMRI exhibited a sensitivity of 93% for detecting clinically significant prostate cancer, markedly outperforming TRUS biopsy, which had a sensitivity of only 48%. Furthermore, mpMRI demonstrated a negative predictive value of 89%, indicating its reliability in ruling out significant cancer and thereby avoiding unnecessary biopsies in approximately 27% of patients.

These results have been further corroborated by studies such as MRI-FIRST and PRECISION II (Table 2), which confirmed that MRI-targeted biopsy strategies detect more clinically significant cancers while reducing the diagnosis of low-grade, indolent tumors. Collectively, the evidence underscores the critical role of mpMRI in reducing overdiagnosis and overtreatment by enabling the more accurate identification of patients who genuinely require intervention, while sparing others from the harms of unnecessary procedures [35].

As a result of this compelling data, mpMRI has been incorporated into major clinical guidelines, including those from the European Association of Urology (EAU), American Urological Association (AUA), and the UK’s National Institute for Health and Care Excellence (NICE), as a recommended pre-biopsy tool in both biopsy-naïve men and those with prior negative biopsies [36]. This paradigm shift represents a move towards more personalized and precise prostate cancer diagnostics, with mpMRI playing a central role in balancing early detection with the imperative to avoid overtreatment.

**Figure 1:**
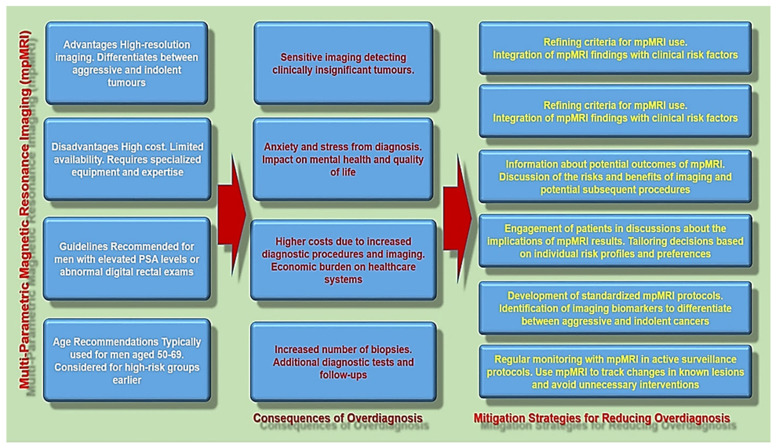
The overview of the mpMRI-associated problems of overdiagnosis and mitigation strategies for reducing it.

Figure 1 provides a summary of the mpMRI-associated problems of overdiagnosis, including the key aspects and possible implications.

Besides this, health system factors such as financial incentives and protective medicine practices can also contribute to the overdiagnosis of PCa. In this regard, new payment models may encourage health care providers to recommend screening tests and procedures regardless of their clinical need. In addition, concerns about malpractice litigation may lead physicians to be cautious and recommend aggressive approaches to diagnosis and treatment, even in cases where watchful waiting or active surveillance may be more appropriate.

## 3. Consequences of Overdiagnosis and Overtreatment of Prostate Cancer

The consequences of the overdiagnosis and overtreatment of PCa are multifaceted [6,37]. Patients may experience unnecessary physical and psychological stress from invasive treatments, while health care systems are burdened with increased costs associated with testing, diagnosis, and treatment. Overdiagnosis and overtreatment also divert resources from more pressing health care needs and can undermine public confidence in cancer screening programs.

Addressing the problem of the overdiagnosis and overtreatment of PCa requires multifaceted approaches [38]. Increasing the specificity of screening tests, such as PSA testing in combination with risk stratification tools, may help identify individuals at the highest risk of aggressive disease. In addition, surveillance strategies such as active surveillance and watchful waiting are increasingly recognized as a viable alternative to immediate treatment for low-risk PCa, minimizing harm while maintaining the ability to intervene when necessary [39]. By implementing strategies to improve risk stratification, promote informed decision-making, and reduce unnecessary interventions, it will be possible to mitigate the harm associated with the overdiagnosis and overtreatment of PCa while optimizing patient outcomes and healthcare resources [40,41].

Identifying aggressive PCa is critical to determining the appropriate treatment strategy and planning [42]. However, distinguishing between indolent and aggressive tumors remain challenging. Current biomarkers, such as the PSA level and Gleason score, have limitations in predicting the aggressiveness and progression of disease. There is a need for new biomarkers that can accurately stratify patients based on the risk of developing metastatic disease or disease recurrence after treatment.

Up to date, imaging techniques such as transrectal ultrasound (TRUS), magnetic resonance imaging (MRI), and positron emission tomography (PET) have improved visualization of the prostate and the detection of suspicious lesions. However, these imaging methods have their limitations, including a low sensitivity and specificity for detecting small or early-stage tumors. In addition, the interpretation of imaging results can be complex and requires knowledge of prostate anatomy and pathology.

The diagnosis of PCa is a complex and multifaceted process, fraught with difficulties. From the limitations of PSA screening to inaccurate biopsy results and a lack of biomarkers for aggressive disease, healthcare providers face numerous barriers to accurately diagnosing and treating PCa. Addressing these challenges requires an interdisciplinary approach that integrates advances in technology, biomarker discovery, and personalized medicine. Table 3 depicts the challenges and corresponding solutions related to the overdiagnosis and overtreatment of PCa.

## 4. Challenges in Treatment and Prognosis of Prostate Cancer

The choice of treatment for PCa depends on various factors, including the stage and aggressiveness of the cancer, the age and general health of the patient, and personal preference [50,51]. The goal of PCa treatment is to remove or destroy cancer cells while minimizing side effects and maintaining quality of life [3]. Before diving into treatment options, it is important to understand the nature of PCa. This type of cancer usually develops slowly and, in many cases, does not require immediate treatment. However, in aggressive forms of the disease, surgery is necessary to prevent it from progressing and spreading to other parts of the body. Treatment options usually include active surveillance, radical prostatectomy, radiation therapy, hormonal therapy, chemotherapy and immunotherapy [52] (Table 4).

Active surveillance involves closely monitoring cancer progression through regular check-ups without immediately starting treatment. It is often recommended for patients with low-risk PCa or people with a limited life expectancy. The advantages of this type of treatment include the ability to avoid the unnecessary side effects associated with treatment. However, this method requires vigilant monitoring, which can cause anxiety and stress for patients, and there is always the risk that the cancer may progress more rapidly than anticipated.

Radical prostatectomy involves the surgical removal of the entire prostate gland and surrounding tissue [53,54]. The advantage of this method is that this type of therapy offers a chance of being cured, especially for localized PCa. Nonetheless, it carries significant risks, including urinary incontinence, erectile dysfunction, and other surgical complications. These potential side effects can have a profound impact on the patient’s quality of life, making the decision to undergo surgery a difficult one.

Another treatment strategy is radiation therapy that uses high-energy X-rays or other forms of radiation to kill cancer cells or suppress their growth [55]. There are several types of radiation therapy: external beam radiation therapy (EBRT) and brachytherapy (internal radiation therapy). The advantages of this type of therapy include the effective treatment of localized PCa. It has fewer side effects compared to surgery. However, radiation therapy can still cause urinary problems, bowel irritation, and erectile dysfunction, posing challenges to maintaining a good quality of life for patients.

Hormone therapy, also known as androgen deprivation therapy (ADT), aims to reduce the levels of male hormones (androgens) that contribute to the growth of PCa [56]. ADT, a well-established form of hormone therapy, functions by suppressing circulating androgen levels or inhibiting androgen receptor activity, thereby limiting the growth and survival of prostate cancer cells. Over the past few decades, ADT has become a fundamental component of PCa management, particularly in advanced and metastatic disease, due to its demonstrated efficacy in controlling tumor progression. The benefits of this type of treatment are that it slows the progression of cancer and relieves symptoms. Nevertheless, it is associated with side effects such as hot flashes, a loss of libido, osteoporosis, and fatigue. These side effects can significantly affect a patient’s daily life and overall well-being, necessitating careful consideration and management.

Currently, ADT is predominantly achieved through pharmacologic manipulation using luteinizing hormone-releasing hormone (LHRH) agonists and antagonists, which effectively reduce circulating testosterone levels to castrate levels [57].

LHRH agonists, such as leuprolide and goserelin, function by initially stimulating LHRH receptors, leading to a transient surge in testosterone, commonly referred to as a “flare”, before downregulating receptor activity and suppressing gonadotropin release [58]. To counteract the potential adverse effects of this initial flare, antiandrogens are often co-administered during the early phase of treatment. In contrast, LHRH antagonists, such as degarelix and the newer oral agent relugolix, bind directly to LHRH receptors without inducing a testosterone surge, thereby providing a more rapid and sustained suppression of androgen levels. Recent clinical data suggest that LHRH antagonists may offer advantages in terms of cardiovascular safety profiles compared to agonists, particularly in patients with pre-existing cardiovascular comorbidities [59,60].

Given the well-documented adverse effects associated with continuous ADT, including metabolic syndrome, bone mineral density loss, cardiovascular risks, and diminished quality of life, intermittent androgen deprivation (IAD) has gained widespread clinical acceptance as a viable alternative strategy. IAD involves cyclic periods of treatment and planned withdrawal, allowing the partial recovery of testosterone levels during off-treatment intervals. This approach has been shown to mitigate some of the long-term toxicities of continuous ADT, such as fatigue, sexual dysfunction, and psychological distress, while maintaining comparable oncologic outcomes in appropriately selected patients with the non-metastatic or biochemical recurrence of PCa.

Chemotherapy is typically used for advanced or metastatic PCa, offering benefits such as the ability to block tumor development and prolong survival. However, this treatment comes with a range of potential side effects, including nausea, hair loss, fatigue, increased susceptibility to infections, and decreased immunity. These side effects can be debilitating and significantly impact the patient’s quality of life.

An alternative strategy for PCa management is immunotherapy, which stimulates the body’s immune system to recognize and attack PCa cells [61]. This approach includes the use of checkpoint inhibitors and therapeutic vaccines. The potential benefits of immunotherapy include long-term responses and fewer systemic side effects compared to traditional treatments. However, its effectiveness can be limited in some patients, and it may cause adverse immune-related events. The variability in patient responses to immunotherapy presents a significant challenge in its application, necessitating further research and personalized treatment approaches. Table 4 summarizes the different treatment options for prostate cancer, highlighting their respective advantages and problems.

**Table 4:** Overview of the different treatment options for prostate cancer.

Treatment Option	Description	Advantages	Problems	Ref.
Active Surveillance	Closely monitoring cancer progression without immediate treatment, often recommended for low-risk PCa or patients with limited life expectancy.	Avoids unnecessary treatment side effects.	Requires close monitoring; may cause anxiety.	[62,63]
Radical Prostatectomy	Surgical removal of the entire prostate gland and surrounding tissue.	Offers a chance of cure, especially for localized PCa.	Risk of urinary incontinence, erectile dysfunction, and other surgical complications.	[64,65]
Radiation Therapy	Uses high-energy X-rays or other forms of radiation to kill or suppress cancer cells. Types include external beam radiation therapy (EBRT) and brachytherapy (internal radiation therapy).	Effective for localized PCa; fewer side effects compared to surgery.	Potential side effects: urinary problems, bowel irritation, erectile dysfunction.	[51,66]
Hormone Therapy (ADT)	Reduces levels of male hormones (androgens) that contribute to the growth of PCa.	Slows cancer progression; relieves symptoms.	Side effects: hot flashes, loss of libido, osteoporosis, fatigue.	[67,68]
Chemotherapy	Uses drugs to kill cancer cells or prevent their proliferation, typically for advanced or metastatic PCa.	Blocks tumor development; prolongs survival.	Potential side effects: nausea, hair loss, fatigue, increased susceptibility to infections, decreased immunity.	[69,70]
Immunotherapy	Stimulates the body’s immune system to recognize and attack PCa cells. Includes checkpoint inhibitors and therapeutic vaccines.	Potential for long-term response; fewer systemic side effects.	Limited effectiveness in some patients; adverse immune-related events.	[71,72]

The treatment and prognosis of prostate cancer involve navigating a complex landscape of options, each with its own set of advantages and challenges. Active surveillance, radical prostatectomy, radiation therapy, hormone therapy, chemotherapy, and immunotherapy each offer potential benefits but also pose significant risks and side effects. Personalized treatment plans, considering the specific characteristics of the cancer and the patient’s overall health and preferences, are essential for optimizing outcomes. Ongoing research and advancements in medical technology continue to improve the efficacy and safety of these treatments, offering hope for the better management of prostate cancer in the future.

## 5. Factors Influencing Treatment Strategy for Prostate Cancer

PCa is a complex disease requiring multiple treatment options, making treatment decisions difficult for both patients and healthcare providers. Several factors influence these decisions, including disease characteristics, patient preferences, and socioeconomic factors. Understanding these factors is essential for tailoring treatment strategies to individual needs and optimizing patient outcomes.

Prostate staging (disease staging), which involves determining the extent and severity of cancer, plays a critical role in treatment decisions for patients with PCa [73]. PCa staging involves assessing the extent of tumor growth, whether the cancer has spread to nearby tissue or distant organs, and other factors that affect prognosis and treatment options. The most widely used staging system for PCa is the TNM system, which classifies tumors based on tumor size (T), lymph node involvement (N), and distant metastasis (M). Another widely used staging system is the Gleason score, which evaluates the aggressiveness of cancer based on a microscopic examination of prostate tissue samples obtained through biopsy.

**Figure 2:**
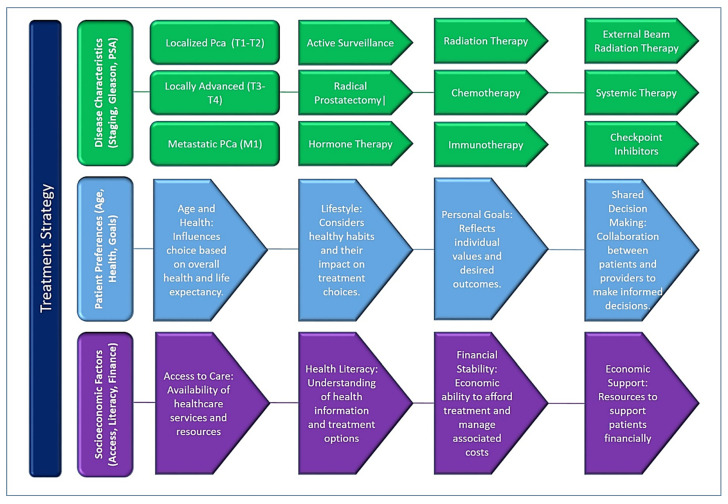
A graphical scheme representing the key factors influencing treatment decisions in prostate cancer, emphasizing the interplay between disease characteristics, patient factors, and healthcare system considerations. Treatment strategies are tailored based on the stage of cancer (localized, locally advanced, or metastatic), Gleason score, PSA levels, PSA density (PSAD), and imaging findings such as multi-parametric MRI (mpMRI). These clinical parameters guide whether patients are managed with active surveillance, surgery, radiation, systemic therapies, or palliative care.

For early-stage localized PCa (T1-T2), treatment options may include active surveillance, surgery (radical prostatectomy), radiation therapy (external beam radiation therapy or brachytherapy), or a combination of these methods [38] (Figure 2). Active surveillance involves closely monitoring the cancer with periodic PSA tests, digital rectal examinations, and repeat biopsies, delaying treatment until there are signs of disease progression. Surgery and radiation therapy are curative treatments aimed at removing or destroying cancer while preserving urinary and sexual function as much as possible.

Stage “locally advanced disease (T3-T4)” refers to a condition in which the cancer has spread beyond the prostate gland, but not to distant organs, and may require more aggressive treatment approaches. Treatment options may include radical prostatectomy with or without adjuvant radiation therapy, external beam radiation therapy with androgen deprivation therapy (ADT), or ADT alone. The goal of treatment is to achieve disease control and prevent the progression of metastatic disease while minimizing treatment-related side effects.

The next stage is metastatic PCa (M1), where the cancer has spread to distant organs such as bones, lymph nodes or other organs, and is usually treated with systemic therapy. Androgen deprivation therapy (ADT), also known as hormone therapy, is the mainstay of treatment for metastatic PCa and aims to suppress testosterone levels and slow cancer growth. Additional treatment options for metastatic disease may include chemotherapy, targeted therapy, immunotherapy, or participation in clinical trials evaluating new treatment approaches.

The stage of PCa plays a critical role in determining treatment outcomes and prognosis for patients [74,75]. Early-stage, localized PCa has a high chance of cure with appropriate treatment, while locally advanced and metastatic disease may require more aggressive therapy to prolong survival and control symptoms. Patients with metastatic PCa have lower overall survival compared with patients with localized disease, highlighting the importance of early detection and treatment.

Another method is the Gleason score, determined by prostate biopsy, which indicates the aggressiveness of PCa [76,77]. Higher Gleason scores indicate more aggressive tumors and may require more aggressive treatment approaches, such as surgery or radiation therapy.

Treatment decisions for PCa are multifaceted and influenced by disease characteristics, patient preferences, and socioeconomic factors. Tailoring treatment strategies to individual needs and preferences is important to optimize patient outcomes and quality of life. Shared decision-making between patients and health care providers, supported by adequate access to health care services and resources, is critical to ensuring treatment decisions are consistent with patients’ goals and preferences. Efforts to eliminate disparities in access to care and improve health literacy can empower patients to make informed decisions and improve PCa outcomes on a broader scale. A graphical scheme representing the factors influencing the treatment strategy for prostate cancer is presented in Figure 2.

The prognosis for PCa varies depending on several factors, including the stage of the cancer at diagnosis, the aggressiveness of the tumor, the effectiveness of treatment, and the patient’s overall health. Early detection and treatment offer the best chance of successful results: the five-year relative survival rate for localized PCa is almost 100%. However, advanced or metastatic PCa may have a poorer prognosis, with lower survival and an increased risk of complications.

Although some risk factors for PCa, such as age and family history, cannot be changed, a healthy lifestyle can help reduce your risk of developing the disease. This includes maintaining a healthy weight, eating a balanced diet rich in fruits, vegetables and whole grains, limiting red meat and processed foods, regular exercise, avoiding smoking and excessive alcohol consumption, and managing stress. Additionally, increasing awareness of PCa, promoting regular screening and early detection, and encouraging open communication between patients and health care providers are essential to reducing the burden of the disease and improving outcomes.

One of strategies for mitigating overdiagnosis and overtreatment in PCa relies on the refinement of PSA Screening Guidelines. The prostate-specific antigen (PSA) test has been a cornerstone of PCa screening. However, its lack of specificity can lead to false positives and unnecessary biopsies. Refining PSA screening guidelines to target high-risk populations more accurately can reduce these issues. Strategies must be focused on the following aspects: (1) adjusting PSA cut-off values based on age to improve specificity, and (2) implementing screening protocols based on individual risk factors such as family history, ethnicity, and genetic predispositions.

Another promising approach to reducing overdiagnosis is the incorporation of novel biomarkers in combination with PSA screening to enhance the accuracy of PCa (PCa) diagnostics [78]. It encompasses PCa Antigen 3 (PCA3), *TMPRSS2-ERG*, Prostate Health Index (phi), SelectMDx, 4Kscore Test and Circulating Tumor Cells (CTCs).

PCa Antigen 3 (PCA3) is a long non-coding RNA that is highly expressed in PCa tissue compared to benign prostate tissue [79,80]. PCA3 is detected through urine-based tests, making it a non-invasive and convenient option for PCa diagnosis. PCA3 testing has demonstrated significant potential in distinguishing between benign prostate conditions and clinically significant PCa, thereby aiding in the decision-making process for prostate biopsy.

The *TMPRSS2-ERG* gene fusion is one of the most common molecular alterations observed in PCa [81,82]. It results from the fusion of the androgen-regulated *TMPRSS2* gene with the *ERG* oncogene, leading to the overexpression of the ERG protein. The detection of *TMPRSS2-ERG* fusion transcripts in urine or tissue samples has shown potential as a biomarker for PCa diagnosis and risk stratification.

The Prostate Health Index (phi) is a blood-based biomarker that combines measurements of total PSA, free PSA, and [-2] proPSA [83]. It provides a more accurate assessment of PCa risk compared to PSA alone, particularly in patients with PSA levels in the gray zone (4–10 ng/mL) [83,84]. The phi test has demonstrated superior performance in distinguishing between aggressive and indolent PCa, thereby guiding treatment decisions and reducing unnecessary biopsies [85].

SelectMDx is a urine-based biomarker test that measures the expression levels of *HOXC6* and *DLX1* genes, which are associated with aggressive PCa [86]. It utilizes a proprietary algorithm to calculate the likelihood of high-grade PCa, helping to identify patients who may benefit from prostate biopsy. SelectMDx has shown promise in reducing unnecessary biopsies while maintaining high sensitivity for clinically significant PCa detection [87].

Circulating Tumor Cells (CTCs) are cancer cells that have shed into the bloodstream from primary tumors or metastatic sites [88]. The enumeration and molecular characterization of CTCs offer valuable insights into the biology of PCa and can serve as a prognostic biomarker for disease progression and treatment response [89]. Additionally, CTC-based liquid biopsies hold potential for the real-time monitoring of treatment efficacy and the detection of treatment-resistant disease. Table 5 provides more detailed information on each above-mentioned biomarker.

Prostate-Specific Antigen Density (PSAD) is a valuable biomarker used in the management of PCa, particularly in helping to determine the need for prostate biopsy [90,91]. PSAD is calculated by dividing the serum level of PSA by the volume of the prostate, which is typically measured using transrectal ultrasound (TRUS) or MRI. PSA is a glycoprotein produced by the epithelial cells of the prostate, and while elevated PSA levels can be indicative of prostate cancer, they can also be raised due to benign prostatic hyperplasia (BPH) or prostatitis. This lack of specificity in PSA alone can lead to unnecessary biopsies or overtreatment in some cases. PSAD refines this by adjusting PSA levels for the prostate size, helping to differentiate between benign prostate conditions and prostate cancer.

**Table 5:** Description of novel PCa biomarkers.

Biomarker	Type	Sample Type	Detection Method	Potential	Clinical Relevance	Ref.
Prostate Cancer Antigen 3 (PCA3)	Long non-coding RNA	Urine	Urine-based assay	Non-invasive, assists in distinguishing between benign and malignant prostate conditions	Moderate	[79]
*TMPRSS2-ERG* Fusion	Gene fusion	Tissue/ Urine	Molecular analysis (PCR)	Aids in diagnosis, prognosis, and risk stratification of prostate cancer	Low to Emerging	[81]
Prostate Health Index (phi)	Protein	Blood	Immunoassay	Improves specificity of PSA testing, aids in identifying aggressive prostate cancer	Moderate to High	[83]
SelectMDx	Gene expression	Urine	mRNA analysis (RT-qPCR)	Identifies patients at risk of clinically significant prostate cancer, reduces unnecessary biopsies	Emerging	[86]
Circulating Tumor Cells (CTCs)	Cancer cells	Blood	Immunocytochemistry	Prognostic biomarker for disease progression, potential for treatment monitoring	Low for early detection	[88]
Prostate-Specific Antigen Density (PSAD)	Antigen	Blood	PSAD calculated by dividing the serum level of prostate-specific antigen (PSA) by the volume of the prostate.	PSAD is a clinical measure used to help assess the risk of PCa.	High	[91]

PSAD is particularly useful for patients whose PSA levels fall into the “grey zone” of 4–10 ng/mL, where the likelihood of cancer is significant but not definitive [92]. In these cases, elevated PSAD values can indicate a higher likelihood of malignancy, while a low PSAD may suggest a lower risk of cancer and potentially reduce the need for a biopsy. PSAD can therefore improve the accuracy of prostate cancer detection and help guide biopsy decisions. For example, a patient with a PSA level above 4 ng/mL but a low PSAD (typically under 0.15 ng/mL/cm^3^) may have a lower risk of having prostate cancer, suggesting that a biopsy might not be immediately necessary. On the other hand, a high PSAD (typically above 0.15 ng/mL/cm^3^) could suggest a higher likelihood of significant malignancy, making a biopsy more advisable.

In addition to its role in guiding biopsy decisions, PSAD can also provide prognostic information [92,93]. Higher PSAD values have been associated with higher Gleason scores and more advanced disease at the time of diagnosis, which can help identify patients who may require more aggressive treatment, such as radical prostatectomy or radiation therapy. PSAD is also valuable in reducing unnecessary biopsies. Since it accounts for prostate size, PSAD can help avoid biopsies in patients with benign prostatic conditions that might otherwise result in false-positive PSA readings. However, while PSAD is a useful tool, it is not without limitations. The accuracy of prostate volume measurement can vary, particularly when using ultrasound, and more precise imaging techniques, such as MRI, are sometimes recommended for better volume assessment. Additionally, PSAD may not be as reliable in certain populations, such as those with very small prostates or those with a history of prostate manipulation. Despite these limitations, PSAD remains a widely accepted and effective marker in the management of prostate cancer. It offers a more nuanced approach to patient care by providing additional insight into the likelihood of cancer and helping to personalize the decision-making process regarding biopsy and treatment.

The 4Kscore Test was developed to overcome the shortcomings of traditional PCa screening methods [94]. It combines the measurement of four kallikrein protein markers in the blood, namely total PSA, free PSA, intact PSA, and human kallikrein 2 (hK2), with clinical information such as age, DRE results, and prior biopsy history [95]. By integrating these variables, the 4Kscore Test provides a personalized risk score indicating the likelihood of finding aggressive PCa on a biopsy. While challenges remain in terms of cost, accessibility, and integration into clinical practice, the potential benefits of the 4Kscore Test in enhancing PCa diagnostics and patient outcomes are substantial.

Apart from biomarkers and PSA screening, mpMRI could be also employed as a valuable tool in the risk stratification of PCa. Conducting mpMRI before biopsy can help to identify areas of concern and reduce the number of unnecessary biopsies. In addition, MRI could be used to guide biopsy needles precisely to suspicious areas, increasing the accuracy of cancer detection (MRI-Targeted Biopsies).

Current research in PCa is increasingly focused on integrating the above-mentioned clinical biomarkers, such as the Prostate-Specific Antigen Density (PSAD), prostate volume, and other parameters, to enhance the pre-biopsy predictive accuracy of imaging modalities like mpMR. While MRI has become a standard tool in the diagnostic workup for patients with elevated PSA levels, its ability to reliably predict clinically significant PCa remains limited, especially in cases of indeterminate lesions [96,97].

According to current guidelines, MRI is recommended before biopsy to help identify suspicious prostate lesions. These lesions are classified using the Prostate Imaging Reporting and Data System (PI-RADS), which ranges from category 1 (very low likelihood of cancer) to category 5 (very high likelihood of cancer) [97,98]. However, the predictive value of intermediate PI-RADS scores is suboptimal. Specifically, lesions categorized as PI-RADS 3 have only about a 25% likelihood of representing clinically significant prostate cancer, while PI-RADS 4 lesions have a predictive value of approximately 50% when confirmed by fusion-targeted biopsies. This means that a substantial proportion of patients undergo unnecessary biopsies or face uncertainty regarding their diagnosis [96,99].

To address these limitations, ongoing research is exploring how biomarkers like PSAD, which adjusts PSA levels for prostate size, can be combined with MRI findings to better stratify patient risk before biopsy. For instance, patients with PI-RADS 3 lesions but low PSAD values may safely avoid biopsy, while those with high PSAD values could be prioritized for further investigation [91]. Similarly, prostate volume measurements are being studied as important factors to contextualize PSA elevations and refine risk assessments.

By integrating such biomarkers with imaging data, researchers aim to develop more accurate and individualized diagnostic pathways that reduce unnecessary biopsies and improve the detection of clinically significant prostate cancer. This multimodal approach holds promise in enhancing the overall precision of prostate cancer diagnostics, optimizing patient care, and reducing the harm of overdiagnosis and overtreatment.

Although the above-mentioned biomarkers (PCA3, *TMPRSS2-ERG* fusion, Prostate Health Index (phi), SelectMDx, and Circulating Tumour Cells) have shown potential in PCa detection, none are currently included in standard clinical guidelines. The main reasons are their limited validation, inconsistent performance across studies, lack of proven impact on clinical outcomes, and cost-effectiveness concerns.

First, large, multi-center, prospective studies are still lacking for many of these biomarkers. Existing evidence often comes from single-center or retrospective studies with limited patient populations, which reduces the generalizability of the results. For example, while PCA3 and SelectMDx can reduce unnecessary biopsies, their sensitivity for detecting clinically significant prostate cancer (csPCa) varies, especially for high-grade tumors, leading to concerns about missed diagnoses.

Second, the added value of these biomarkers over established tools like PSA, PSAD, and MRI remains unclear. MRI is already widely adopted and provides valuable anatomical and functional information. To be incorporated into guidelines, biomarkers must demonstrate a clear improvement in diagnostic accuracy, ideally in combination with MRI, and show they can meaningfully influence clinical decision-making. So far, the incremental benefit of biomarkers has not been compelling enough to change standard practice.

Third, cost-effectiveness is a major consideration. Many of these tests involve complex molecular analyses, adding financial and logistical burdens to healthcare systems. Without strong evidence that they improve patient outcomes or reduce overall costs by avoiding unnecessary procedures, their widespread adoption remains unlikely. Lastly, regulatory approval and guideline inclusion require robust, reproducible evidence that a biomarker improves patient care, not just diagnostic accuracy. Until such evidence is available, current guidelines remain cautious, focusing on PSA, PSAD, and MRI, which have a well-established role in PCa management.

Genomic testing can also provide insights into the aggressiveness of PCa, aiding in risk stratification. For example, the Oncotype DX Genomic Prostate Score might be harnessed for evaluating gene expression to predict disease aggressiveness [100,101]. In addition, the Decipher Test can also help with analyzing genomic data to inform treatment decisions and predict the risk of metastasis [102,103].

Recent advances in molecular oncology have highlighted the potential use of non-coding RNAs (ncRNAs), particularly microRNAs (miRNAs) and long non-coding RNAs (lncRNAs), as valuable biomarkers for PCa [104] (Table 6). Unlike traditional protein-coding genes, ncRNAs regulate gene expression at various levels and play critical roles in cancer initiation, progression, and metastasis. Their stability in body fluids and high specificity to tumor biology make them promising candidates for non-invasive diagnostic and prognostic applications [105]. In the context of prostate cancer, overdiagnosis and overtreatment remain significant challenges, largely due to the limited specificity of current screening methods such as PSA testing and mpMRI. Many detected tumors are indolent and unlikely to impact patient survival, yet they often lead to unnecessary biopsies and aggressive treatments with substantial morbidity. Incorporating ncRNA-based biomarkers offers a novel strategy to refine risk stratification, enabling clinicians to distinguish between clinically significant and insignificant tumors more accurately [106,107].

MicroRNAs (miRNAs), small ncRNAs that post-transcriptionally regulate gene expression, have been extensively studied in PCa (Table 6). Specific miRNA expression profiles are associated with tumor aggressiveness, progression, and therapeutic resistance. For instance, miR-141, miR-21, and miR-375 have shown potential as biomarkers for diagnosis and prognosis, correlating with the disease stage and Gleason score. Their detection in blood, urine, or tissue samples allows for the minimally invasive assessment of tumor behavior, supporting more informed clinical decisions.

Long non-coding RNAs (lncRNAs), such as PCA3 (prostate cancer antigen 3) and *SChLAP1* (Second Chromosome Locus Associated with Prostate-1), have also demonstrated diagnostic and prognostic relevance [106]. PCA3, detectable in post-DRE urine samples, has already been clinically validated to reduce unnecessary biopsies in men with elevated PSA levels but a low risk of aggressive disease. Emerging lncRNAs like PCAT-1 and MALAT1 are being investigated for their roles in predicting disease progression and treatment outcomes.

The integration of ncRNA biomarkers into clinical workflows has the potential to complement existing diagnostic tools and improve patient selection for biopsy and treatment. By identifying molecular signatures indicative of aggressive disease, clinicians can prioritize interventions for patients who are most likely to benefit, while sparing low-risk individuals from invasive procedures and overtreatment. Moreover, ncRNAs may enhance the predictive value of imaging modalities like mpMRI and augment multiparametric risk models that combine PSA, PSAD, prostate volume, and genomic data.

However, despite promising research, several challenges must be overcome before their widespread clinical adoption. The standardization of assay methods, their validation in large, diverse patient cohorts, and the demonstration of cost-effectiveness are essential to ensure the clinical utility of ncRNA-based tests. Regulatory approval and incorporation into clinical guidelines will also require robust evidence of improved patient outcomes.

## 6. Prostate Enlargement and Cancer Risk: The Protective Hypothesis and Its Reflection in PSAD

Recent research has brought renewed attention to the hypothesis that prostate size, particularly in the setting of benign prostatic hyperplasia (BPH), may confer a protective effect against the development of PCa [117,118]. Historically, an enlarged prostate was primarily viewed as a diagnostic challenge, often complicating cancer detection by elevating PSA levels and making biopsies technically more difficult. However, accumulating evidence now suggests a more nuanced relationship, wherein a larger prostate size is inversely associated with the incidence and detection of clinically significant prostate cancer (csPCa) [119] (Table 7).

Multiple epidemiological and imaging studies have consistently demonstrated that men with larger prostate volumes have a lower risk of harboring csPCa compared to those with smaller glands [120]. This inverse association is thought to be more than a statistical artefact and has gained biological plausibility through several proposed mechanisms. Anatomically, prostate cancer predominantly originates in the peripheral zone, while BPH involves the hyperplastic growth of the transition zone. As the transition zone enlarges with age, it may compress, displace, or alter the microenvironment of the peripheral zone, thereby reducing the available space and possibly impeding the carcinogenic process in these regions. Furthermore, an increased glandular volume may be associated with altered vascularization, stromal–epithelial interactions, and the hormonal milieu, which could influence carcinogenesis.

Another key aspect reflecting this inverse relationship is the Prostate-Specific Antigen Density (PSAD), a well-established biomarker for prostate cancer risk stratification [121]. PSAD is calculated by dividing the serum PSA by the prostate volume, effectively normalizing PSA levels to the gland size (Table 7). In clinical practice, PSAD helps differentiate between PSA elevations due to benign enlargement and those potentially driven by malignancy. A lower PSAD in men with large prostates typically indicate a lower probability of csPCa, thereby refining biopsy decisions. This reinforces the clinical relevance of prostate size not merely as a confounding factor but as an integral variable in prostate cancer risk models.

The growing body of evidence supporting the protective hypothesis regarding a larger prostate size has important implications for patient management (Table 7). Incorporating the prostate volume into predictive nomograms, alongside PSA levels, MRI findings, and other clinical parameters, can improve diagnostic accuracy, reduce unnecessary biopsies, and limit the overdiagnosis of indolent cancers. Particularly in men with PSA levels in the so-called “grey zone” (4–10 ng/mL), prostate size and PSAD provide valuable additional information to guide clinical decisions [122].

However, it is important to exercise caution in interpreting these findings. While an inverse association exists, prostate size alone cannot be regarded as an independent protective factor. Prostate cancer is a multifactorial disease influenced by genetic predisposition, age, race, lifestyle, hormonal factors, and molecular alterations. Additionally, a smaller prostate size may not only correlate with a higher incidence of cancer but also with more aggressive tumor biology, as suggested by some studies. Therefore, the prostate volume should be considered as part of a comprehensive risk assessment strategy rather than as a standalone determinant of cancer risk. In conclusion, the hypothesis that a larger prostate size may reduce the risk of clinically significant prostate cancer has gained traction through recent research, offering both biological rationale and clinical relevance. This concept is reflected in the widespread use of PSAD, which accounts for the prostate volume in risk stratification models.

Integrating prostate size into diagnostic algorithms enhances the precision of prostate cancer detection and supports a more individualized approach to patient care. Ongoing prospective studies and validation in diverse populations are essential to further establish the role of prostate volume in prostate cancer risk assessment and clinical decision-making. Table 6 provides a comparison of traditional approaches vs. emerging new strategies for PCa diagnostics.

**Table 7:** Comparison of traditional methods vs. emerging strategies for prostate cancer diagnostics.

Aspect	Traditional Methods (PSA + Random Biopsy)	MRI	Biomarkers	Targeted Biopsy	Ref.
Screening Tool	PSA test: blood-based, non-specific, high false positives/negatives.	mpMRI: Non-invasive imaging for lesion localization and risk assessment.	Blood/urine tests (phi, 4Kscore, PCA3, SelectMDx) increase specificity for csPCa.	Used after MRI to sample MRI-visible lesions. Not a screening tool itself.	[11,123]
Biopsy Approach	Random TRUS-guided biopsy: blind, systematic sampling of 10–12 cores.	Identifies suspicious regions before biopsy, guides biopsy decision.	Supports biopsy decision-making by risk stratification.	MRI-TRUS fusion, cognitive, or in-bore MRI-guided biopsy targeting MRI-visible lesions.	[124,125]
Sensitivity and Specificity	PSA: variable sensitivity (21–68%), low specificity, leads to unnecessary biopsies.	High sensitivity (~88%) and specificity (~74%) for csPCa.	Improves specificity, differentiating indolent vs. aggressive cancers.	Higher detection rate of csPCa, fewer missed lesions compared to random biopsy.	[126,127,128]
Overdiagnosis and Overtreatment	High risk of detecting insignificant cancers, leading to overtreatment.	Reduces detection of indolent lesions, focuses on csPCa.	Helps avoid unnecessary biopsies and treatment of indolent cancers.	Targets clinically significant lesions, reduces overdiagnosis.	[129,130]
Invasiveness and Complications	Invasive biopsy with risk of infection, bleeding, urinary retention.	Non-invasive imaging modality.	Minimally invasive (blood/urine samples).	Fewer biopsy cores, lower complication rates compared to random biopsy.	[131,132]
Cost and Accessibility	PSA: low-cost, random biopsy widely available but resource-intensive.	High cost, limited access in some regions, requires radiology expertise.	Variable cost, availability increasing, simpler than MRI.	Requires specialized equipment and expertise for MRI-guided targeting.	[9,133]
Clinical Guidelines and Adoption	PSA + random biopsy remains standard but controversial.	Recommended pre-biopsy tool in AUA, EAU guidelines for elevated PSA or prior negative biopsy.	Used as adjuncts for better risk assessment and biopsy decision-making.	Increasingly preferred for biopsy guidance after MRI in guidelines.	[134,135]
Limitations	PSA lacks specificity; random biopsy may miss csPCa; overdiagnosis common.	Operator-dependent interpretation; variability in MRI quality and reporting.	Lack of universal thresholds; not all biomarkers are widely validated.	Access to technology and expertise can be limited; requires MRI integration.	[136,137]

## 7. PCa Overtreatment: Trends and Perspectives

Over the past two decades, the management of PCa has undergone a profound transformation, largely driven by efforts to mitigate the overtreatment of low-risk disease. In the early 2000s, prostate cancer diagnosis was heavily reliant on prostate-specific antigen (PSA) screening followed by systematic random biopsies. This approach led to the widespread detection of indolent, low-grade tumors that were unlikely to progress or impact life expectancy. Nevertheless, the prevailing clinical practice at the time favored aggressive interventions. Data from that period indicate that over 90% of men with low-risk prostate cancer were treated with radical prostatectomy or radiotherapy, despite the limited oncological benefit in such cases and the significant risk of treatment-related morbidity, including urinary incontinence, erectile dysfunction, and bowel dysfunction [138,139].

The recognition of these harms, coupled with increasing evidence that many prostate cancers follow an indolent course, sparked a critical reassessment of management strategies. Pivotal studies, such as the PIVOT trial (2012) and long-term observational cohorts, demonstrated that the immediate radical treatment of low-risk prostate cancer offered minimal survival benefits compared to conservative approaches [140]. In parallel, the concept of active surveillance (AS) gained traction as a safe alternative to immediate treatment. Active surveillance involves regular monitoring through PSA testing, digital rectal examination, mpMRI, and repeat biopsies, with curative treatment deferred until signs of disease progression emerge.

The incorporation of active surveillance into clinical guidelines represented a major shift in practice. By the mid-2010s, authoritative bodies such as the National Comprehensive Cancer Network (NCCN), American Urological Association (AUA), and European Association of Urology (EAU) began to recommend this as the preferred initial management strategy for men with low-risk, localized prostate cancer [141]. These guidelines emphasized risk stratification and the importance of avoiding overtreatment in cases where the disease was unlikely to pose a threat to the patient’s health.

The impact of these guideline changes is evident in real-world practice. In the United States, for example, the proportion of men with low-risk prostate cancer managed with active surveillance increased dramatically from approximately 10% in 2005 to nearly 60% by 2019 [142,143]. Similar upward trends have been reported in Canada, the Netherlands, Sweden, and the United Kingdom, where national initiatives and patient education campaigns further promoted the adoption of AS. Concurrently, the rate of radical prostatectomy for low-risk prostate cancer has seen a substantial decline, with studies indicating a reduction of up to 50% in surgical overtreatment over the past decade.

Several factors have contributed to this positive trend. Advances in diagnostic imaging, particularly the widespread implementation of mpMRI, have improved the ability to distinguish clinically significant from indolent disease, reducing the likelihood of unnecessary biopsies and interventions. Furthermore, the use of genomic and molecular biomarkers has enhanced risk stratification, aiding clinicians and patients in making informed management decisions.

Importantly, these changes reflect not only technological advancements but also a broader cultural shift towards shared decision-making and patient-centered care. Patients are now more actively involved in their treatment choices, with an increased awareness of the potential harms of overtreatment and the safety of surveillance strategies.

Despite this progress, challenges remain. Variability in the uptake of active surveillance persists across different healthcare systems, regions, and demographic groups. Factors such as physician preference, healthcare access, patient anxiety, and socioeconomic disparities continue to influence treatment decisions. Nonetheless, the overall temporal trend over the past 10–20 years unequivocally demonstrates a significant reduction in the overtreatment of prostate cancer, driven by evolving evidence, updated guidelines, and the integration of precision diagnostic tools.

While the problem of prostate cancer overtreatment has been extensively studied in high-income countries, its relevance in developing countries remains an important yet underexplored issue. In many low- and middle-income countries (LMICs), prostate cancer is increasingly recognized as a significant public health concern due to the rising life expectancy and broader adoption of PSA testing [144]. However, limited access to advanced diagnostic technologies, such as mpMRI and novel molecular biomarkers, poses substantial challenges to accurate risk stratification and appropriate treatment selection [145].

In these settings, prostate cancer diagnosis often relies heavily on PSA levels and systematic random biopsies, similar to the outdated approaches previously used in wealthier nations. This reliance increases the risk of overdiagnosing indolent, low-risk tumors that may never progress to clinical significance. Without reliable tools to differentiate aggressive cancers from harmless ones, clinicians may lean towards radical treatments, including surgery and radiotherapy, as a precautionary measure. Consequently, overtreatment becomes a hidden but critical problem, leading to the unnecessary exposure of patients to treatment-related morbidities that can severely affect their quality of life [146].

Compounding this issue is the variability in healthcare infrastructure, expertise, and the availability of multidisciplinary care teams in LMICs [147]. In many regions, the lack of standardized prostate cancer management guidelines adapted to local resources results in inconsistent clinical practices. Furthermore, cultural factors, limited patient education, and economic constraints often influence decision-making, sometimes prioritizing immediate treatment over active surveillance, even when the latter would be oncologically safe.

Unlike high-income countries, where active surveillance adoption has been driven by robust clinical evidence and patient-centered care models, developing countries face hurdles in implementing such strategies [148]. With regular monitoring through PSA testing, imaging, and repeat biopsies, the core components of active surveillance may be impractical due to limited resources, fragmented healthcare delivery, and patient follow-up challenges. This practical reality often shifts the risk–benefit balance, making active surveillance underutilized, despite its potential to reduce overtreatment.

Nevertheless, some positive developments are emerging. The gradual dissemination of international guidelines, a growing awareness of overtreatment harms, and the introduction of cost-effective diagnostic innovations are slowly changing the landscape. Efforts to implement simplified risk stratification tools, promote education among healthcare providers, and develop affordable imaging alternatives (such as bi-parametric MRI or ultrasound-based techniques) hold promise in improving diagnostic accuracy while curbing overtreatment rates.

To summarize, the past two decades have marked a paradigm shift in the management of low-risk prostate cancer, with a substantial move away from blanket aggressive treatment towards more nuanced, individualized care. This shift has led to a meaningful decrease in overtreatment rates, minimizing harm to patients while maintaining oncological safety.

## 8. Conclusions

PCa remains a complex and prevalent malignancy where the benefits of early detection through PSA-based screening must be weighed against the risks of overdiagnosis and overtreatment. This review highlights that while PSA testing has contributed significantly to early diagnosis, it has also led to the detection and often unnecessary treatment of indolent tumors, which pose a minimal risk to patients’ longevity or quality of life.

The review demonstrates that overtreatment frequently results in physical, psychological, and economic burdens, including urinary incontinence, erectile dysfunction, patient anxiety, and strain on healthcare resources. These outcomes necessitate a shift in clinical practice toward more nuanced and personalized approaches. The incorporation of mpMRI as a standard pre-biopsy triage tool has shown considerable promise in enhancing diagnostic specificity and reducing unnecessary biopsies and treatments.

Active surveillance has emerged as a cornerstone strategy in managing low-risk PCa, supported by robust evidence and international guidelines. Novel biomarkers, such as PCA3, phi, *TMPRSS2-ERG*, SelectMDx, and PSAD, are increasingly being explored for their potential to refine risk stratification and reduce unnecessary interventions. Despite their promise, many of these biomarkers are yet to be fully validated and integrated into standard clinical workflows due to issues of cost, reproducibility, and limited outcome data.

Emerging evidence also supports the hypothesis of a larger prostate size protecting against clinically significant cancer, further emphasizing the role of prostate volume and PSA density in risk modelling. Advances in non-coding RNA biomarkers and genomics likewise hold potential for the more accurate prediction of tumor behaviour and therapy response, although broader clinical adoption is constrained by technological and regulatory challenges.

Globally, the trend toward reducing overtreatment is more apparent in high-income settings due to access to advanced diagnostics and patient-centered care models. However, in low- and middle-income countries, barriers such as limited access to imaging, biomarker testing, and follow-up infrastructure continue to fuel overtreatment, underlining the need for context-appropriate solutions.

In conclusion, mitigating overdiagnosis and overtreatment in prostate cancer requires a paradigm shift toward precision medicine, involving risk-adapted screening, imaging-guided diagnostics, biomarker-informed stratification, and shared decision-making. As research evolves and technologies mature, healthcare systems must adapt by fostering guideline updates, equitable access, and public education to ensure that PCa care is both effective and minimally harmful.

## Figures and Tables

**Table 1:** An overview of the current issues of overdiagnosis and overtreatment in prostate cancer.

Aspect	Description	Potential Risks of Inaction	Patient Outcomes and Long-Term Effects	Ethical Considerations	Global Perspectives	Ref.
Definition of Overdiagnosis	Detection of PCa through screening that would not have caused symptoms or death during a patient’s lifetime.	Continued unnecessary diagnoses leading to overburdened healthcare systems.	Increased anxiety and potential for overtreatment.	Ethical dilemma of informing patients about non-threatening cancers.	Some countries have restricted PSA testing to high-risk individuals.	[9]
Causes of Overdiagnosis	Widespread use of PSA testing, increased imaging, detection of slow-growing cancers.	Rising false positives and unnecessary treatments.	Psychological burden and unnecessary treatments.	Challenge of balancing early detection with avoiding overtreatment.	Countries like Sweden emphasize risk-adapted screening.	[10]
Impact of Overdiagnosis	Psychological distress, unnecessary medical consultations, strain on healthcare resources.	Wasted resources, leading to fewer available funds for life-threatening conditions.	Potential decline in trust in medical recommendations.	Ethical responsibility of physicians to ensure patients are not harmed by overdiagnosis.	Countries adopting stricter screening criteria.	[11]
Definition of Overtreatment	Treatment of prostate cancer that would not have progressed or caused harm if left untreated.	Increased rates of avoidable complications and reduced quality of life.	Unnecessary exposure to treatment risks and lifelong side effects.	Ethical concerns regarding patient autonomy in treatment decisions.	Some nations promote active surveillance as a first-line strategy.	[12]
Causes of Overtreatment	Inability to distinguish aggressive from indolent cancers, patient and physician preference for active treatment.	Overuse of aggressive treatments leading to resource depletion.	Complications such as incontinence and erectile dysfunction.	Patient pressure to “do something” despite low risk.	The UK and Canada emphasize shared decision-making in treatment.	[13]
Impact of Overtreatment	Treatment-related side effects, reduced quality of life, financial burden.	Healthcare systems burdened with avoidable interventions.	Increased economic and emotional stress on patients.	Ethical tension between providing treatment vs. potential harm.	European guidelines encourage more conservative approaches.	[12]
Strategies to Mitigate Overdiagnosis	Risk-based screening, use of biomarkers and imaging, patient education.	Continued unnecessary treatment and financial strain.	Lower burden of unnecessary diagnoses when applied correctly.	Ensuring informed patient choices without undue influence.	Countries adopting multi-parametric MRI for risk assessment.	[12]
Strategies to Mitigate Overtreatment	Active surveillance, personalized treatment plans, shared decision-making.	Unchecked increase in unnecessary treatments.	Reduction in treatment-related morbidity when applied correctly.	Respecting patients’ preferences while ensuring medical necessity.	Adoption of less invasive treatments globally.	[14]
Role of Active Surveillance	Monitoring low-risk cancer progression through periodic testing.	Delayed detection of aggressive cases if not properly managed.	Reduced need for immediate intervention, preserving quality of life.	Ethical challenge of balancing risks of waiting vs. acting too soon.	Widely adopted in European healthcare systems.	[15]
Importance of Genetic Testing	Identifying mutations (e.g., BRCA1, BRCA2) to assess risk.	Failure to incorporate genetic risk can lead to mismanagement of cases.	More accurate treatment decisions and improved patient outcomes.	Ethical concerns regarding genetic discrimination.	Countries developing genetic databases for better risk profiling.	[16]
Research and Policy Recommendations	Promoting biomarker research, revising screening guidelines.	Lack of innovation in screening may lead to continued overdiagnosis.	Evidence-based policies can enhance patient care.	Ensuring policies align with patient safety and informed choice.	Nations integrating genetic research into clinical practice.	[17]
Patient Education and Awareness	Providing clear information on screening risks and benefits.	Patients making uninformed decisions leading to unnecessary treatment.	Better patient engagement and reduced anxiety over low-risk cancers.	Ethical responsibility to provide unbiased and transparent information.	National campaigns promoting informed decision-making.	[18]
Technological Advancements	AI-driven imaging, molecular diagnostics, liquid biopsy research.	Stagnation in diagnostic progress leading to persistent challenges.	Improved accuracy in early detection and risk stratification.	Ethical issues surrounding AI in medical decision-making.	Countries adopting AI-based tools in radiology.	[19]

**Table 2:** Evidence from Key Clinical Trials.

Study	Population (*n*)	Avoided Biopsies	csPCa Detection	Reduction of Insignificant Cancer
PRECISION	500	28% avoided biopsy	38% (MRI-targeted) vs. 26% (TRUS)	9% (MRI) vs. 22% (TRUS)
PROMIS	576	Up to 27% biopsies avoided	Sensitivity 93% (MRI) vs. 48% (TRUS)	High NPV (89%) reduced unnecessary biopsy
MRI-FIRST	251	Confirmed MRI triage value	More csPCa detected with MRI-targeted biopsy	Fewer low-grade cancers diagnosed

**Table 3:** Challenges and solutions related to the overdiagnosis and overtreatment of PCa.

Category	Challenges	Proposed Solutions	Ref.
Consequences of Overdiagnosis and Overtreatment	Unnecessary physical and psychological distress from invasive treatments	Enhance screening specificity with PSA testing combined with risk stratification tools	[17]
	Increased healthcare costs for testing, diagnosis, and treatment	Implement active surveillance and watchful waiting for low-risk cases	[43]
	Resource diversion from more critical healthcare needs	Promote shared decision-making to reduce unnecessary interventions	[44]
	Erosion of public confidence in cancer screening programs	Education and public campaigns to improve an awareness about PCa screening	[45]
Challenges in Identifying Aggressive PCa	Difficulty differentiating between indolent and aggressive tumors	Develop next-generation biomarkers for accurate risk stratification	[17]
	PSA levels and Gleason scores have limited accuracy in predicting disease progression	Introduce molecular and genetic profiling for better classification of aggressive cases	[2]
	Lack of precise biomarkers for assessing metastatic risk and recurrence	Research on new biomarkers for assessing metastatic risk and PCs recurrence	[17]
Limitations of Imaging Techniques	Conventional imaging methods (TRUS, MRI, PET) have low sensitivity and specificity for detecting small or early-stage tumors	Improve imaging technologies with AI-assisted interpretation	[46]
	Interpretation of imaging results requires specialized expertise in prostate anatomy and pathology	Standardize diagnostic protocols to enhance accuracy and reduce variability among radiologists	[47]
Complexity of PCa Diagnosis	PSA-based screening has limitations in specificity	Adopt an interdisciplinary approach integrating technological advancements, biomarker research, and personalized medicine	[48]
	High risk of inaccurate biopsy results		[44]
	Lack of definitive diagnostic tools for aggressive disease		[49]

**Table 6:** Summary of the clinical relevance, challenges, and solutions regarding the use of non-coding RNAs (ncRNAs) as biomarkers in prostate cancer.

Clinical Relevance	Challenges	Solutions	Ref.
Improved risk stratification: ncRNAs (miRNAs, lncRNAs) help distinguish aggressive from indolent PCa, reducing overtreatment.	Lack of assay standardization and reproducibility across labs and platforms.	Develop and implement international guidelines for ncRNA assay protocols and quality control.	[108,109]
Non-invasive diagnostics: Detection of ncRNAs in blood/urine (e.g., PCA3, miR-141) allows for less invasive testing and fewer unnecessary biopsies.	Limited validation in large, multi-ethnic, and prospective cohorts.	Need in the multi-centre, prospective studies including diverse populations to validate biomarker performance.	[110,111]
Prognostic value: Specific ncRNAs (e.g., miR-21, SChLAP1) correlate with tumor aggressiveness, recurrence risk, and therapy resistance.	Insufficient integration with existing clinical risk models and lack of consensus on cut-off values.	Integrate ncRNA panels into multiparametric risk models and establish clinically relevant thresholds.	[112,113]
Therapeutic guidance: ncRNA profiles can inform personalized treatment decisions and predict response to therapy.	High cost and limited accessibility of advanced molecular testing in routine practice.	Invest in technology transfer, cost reduction strategies, and reimbursement frameworks for ncRNA assays.	[112,114]
Complementary to imaging: ncRNAs can enhance the specificity of mpMRI and PSA-based screening, improving patient selection.	Regulatory and guideline adoption lag behind emerging evidence.	Generate robust outcome data and advocate for inclusion in clinical guidelines (e.g., NCCN, EAU, AUA).	[115,116]

## Data Availability

Not applicable.
